# Food-Web Models Predict Species Abundances in Response to Habitat Change

**DOI:** 10.1371/journal.pbio.0040324

**Published:** 2006-09-26

**Authors:** Nicholas J Gotelli, Aaron M Ellison

**Affiliations:** 1 Department of Biology, University of Vermont Burlington, Burlington, Vermont, United States of America; 2 Harvard Forest, Harvard University, Petersham, Massachusetts, United States of America; University of Florida, United States of America

## Abstract

Plant and animal population sizes inevitably change following habitat loss, but the mechanisms underlying these changes are poorly understood. We experimentally altered habitat volume and eliminated top trophic levels of the food web of invertebrates that inhabit rain-filled leaves of the carnivorous pitcher plant Sarracenia purpurea. Path models that incorporated food-web structure better predicted population sizes of food-web constituents than did simple keystone species models, models that included only autecological responses to habitat volume, or models including both food-web structure and habitat volume. These results provide the first experimental confirmation that trophic structure can determine species abundances in the face of habitat loss.

## Abstract 2

Manipulating habitat volume and the presence of top trophic levels of an aquatic community reveals that trophic structure can determine species abundance despite habitat loss.

## Introduction

The loss of natural habitat area often is accompanied by the disappearance of large-bodied top predators and the upper trophic levels of food webs [1–3]. However, several pieces of evidence suggest that habitat area alone may be insufficient to predict changes in population size. Predictions of ecological models [4,5], patterns of food-web structure in small versus large habitat fragments [6], and recent observations of collapsing island communities [1,7] all suggest that trophic interactions must be considered in order to predict how abundances of populations will change in the face of habitat loss and alteration. However, existing tests of the role of trophic interactions in determining species abundances as habitats contract are correlative only. In this study, we provide the first evidence from a controlled field experiment for the importance of trophic structure in controlling abundances of multiple species in an aquatic food web. Moreover, we demonstrate that models of trophic structure account for the results better than do simpler models that focus only on responses of individual species to changes in habitat size or structure, or models that include both food-web structure and habitat volume.

For multitrophic assemblages, two broad classes of community models predict the potential responses of populations to habitat change. (1) Single-factor models emphasize the unique responses of individual species to variation in habitat area. This framework includes island biogeographic models [8], as well as single-species demographic analyses [9] and assessments of extinction risk. Single-factor models also include keystone species effects, which emphasize responses of populations to changes in the abundance of a single keystone species, such as a top predator [10,11]. This framework includes much current research on habitat alterations by foundation species [12] and ecosystem engineers [13]. (2) Food-web models emphasize the shifts in abundance that result from multiple trophic interactions and the transfer of energy and biomass through a food web. This framework includes top-down and bottom-up processes [14], trophic cascades [15], and more complex interactions across multiple trophic levels [16].

Unfortunately, published studies of the effects of habitat contraction have relied on conventional analyses that do not explicitly compare these alternative frameworks [17,18]. Although analysis of variance and other statistical protocols can quantify community change, they cannot be used to distinguish between simple responses of species to habitat contraction (single-factor volume model) and more complex responses to changes in the abundance of other species (food-web and keystone species models). A third possibility is that trophic responses dominate the responses, even in the face of habitat alterations. In this study, we used realistic field manipulations of habitat volume and removal of top trophic levels of entire aquatic communities. These manipulations induced major alterations in habitat size and community structure that have been studied previously in nonexperimental settings [1]. For the first time, we have experimentally assessed the relative importance of autecological responses, keystone species effects, and trophic interactions in accounting for changes in species' abundance.

## Results

### The Aquatic Food Web of *Sarracenia*

**Figure 1:**
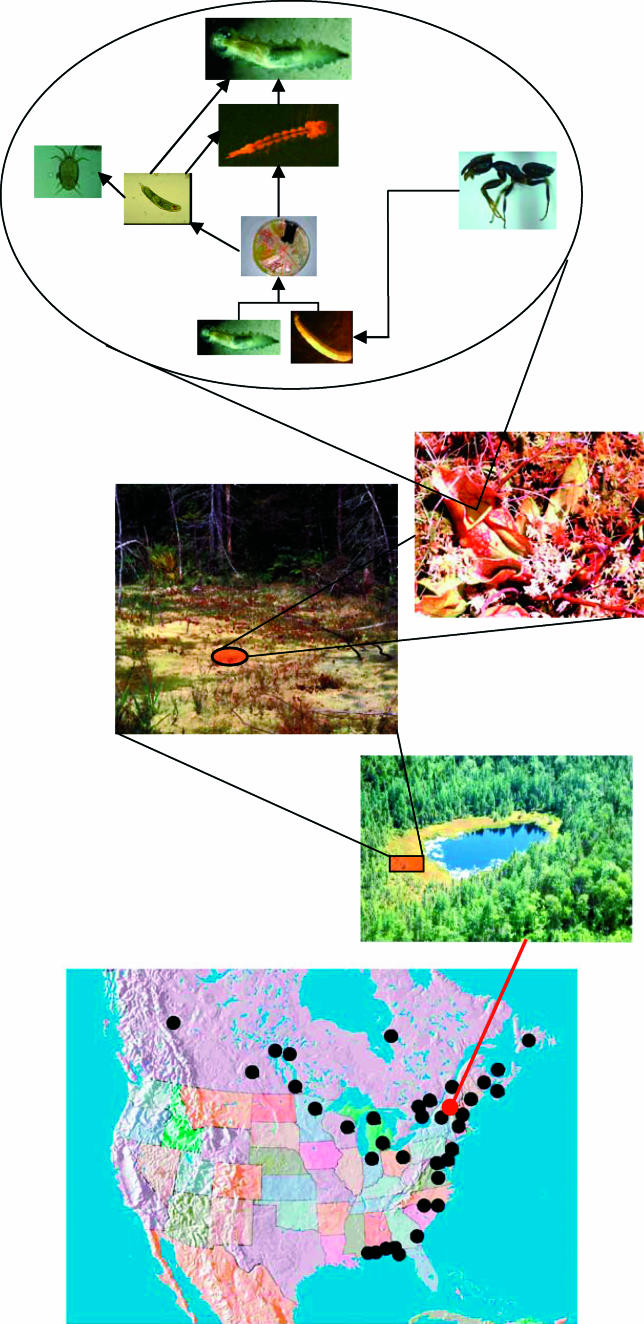
The *Sarracenia* Food Web Each leaf of the northern pitcher plant Sarracenia purpurea contains an entire aquatic food web, with a resource base consisting of captured arthropod prey. *Sarracenia* occurs in *Sphagnum* bogs and seepage swamps throughout the eastern United States and Canada (sites at which similar species assemblages can be found [27] are shown as black dots). (Photo Montage: Aaron M. Ellison)

The macroinvertebrate community associated with the northern pitcher plant Sarracenia purpurea (Figure 1) is a model system for testing mechanisms controlling abundance in the face of habitat change [19]. S. purpurea is a long-lived perennial plant that grows in peat bogs and seepage swamps throughout southern Canada and the eastern United States [20]. The plant grows as a rosette and produces a set of six to 12 new tubular leaves each year. During the growing season, leaves open approximately every 17 d and fill with rain water; an aquatic food web quickly develops in these water-filled leaves [21]. Leaves are photosynthetically most active in their first year, but persist, capture prey, and are used as macroinvertebrate habitat for 1 to 2 y [22]. The base of the food web is captured arthropod prey (predominantly ants and flies), which is shredded and partially consumed by midge *(Metriocnemus knabi)* and sarcophagid fly *(Fletcherimyia fletcheri)* larvae [23]. Shredded prey are then processed by a subweb of bacteria and protozoa [24], which respectively are prey to filter-feeding rotifers *(Habrotrocha rosi)* and mites *(Sarraceniopus gibsonii)*. Larvae of the pitcher plant mosquito Wyeomyia smithii feed on bacteria, protozoa, and rotifers [25]. Large (third instar) larvae of F. fletcheri feed on rotifers and small (first and second instar) larvae of W. smithii [26]. Thus, the *Sarracenia* food web exhibits the same complex linkages across multiple trophic levels that characterize other aquatic and terrestrial food webs [16]. Furthermore, the same assemblage of macroinvertebrate species can be found associated with S. purpurea throughout its broad geographic range—from the Florida panhandle to Labrador and west to the Canadian Rocky Mountains [27].

In a replicated field experiment, we simultaneously manipulated habitat volume (adding or removing water from leaves) and simplified the trophic structure of the *Sarracenia* food web (by retaining or removing larvae of all three dipterans: *Metriocnemus*, *Wyeomyia,* and *Fletcherimyia*). We measured the abundance of the resident species in each replicate leaf (Dataset S1) and compared the fit of food-web models, keystone species models, and autoecological response models to the data (see Materials and Methods).

### Model Fit Statistics

**Figure 2:**
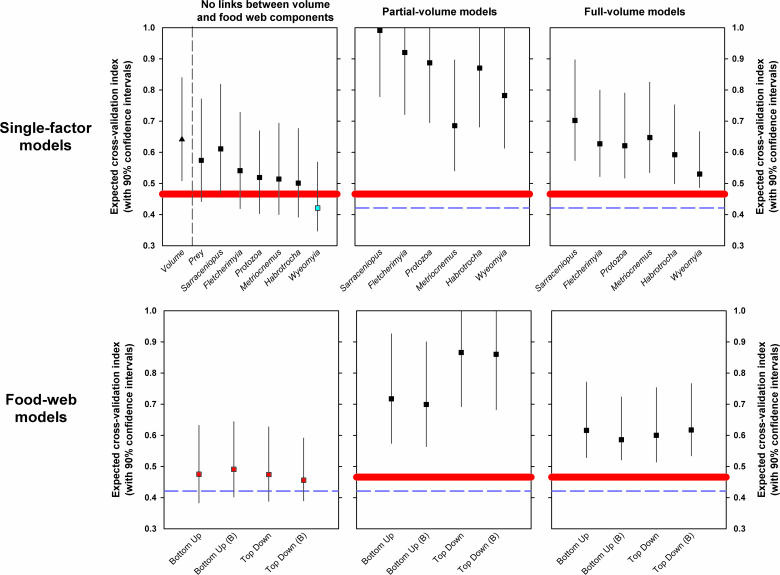
Cross-Validation Indices and 90% Confidence Intervals for Path Analysis Models of Macroinvertebrate Abundance The smaller the index, the better is the fit of the data to the predictions of the model. The upper row of panels includes all single-factor models (Tables S3 to S6). The single-factor model that has only habitat volume as a predictor variable (see Table S3) is indicated by a triangle to the left of the dashed vertical line. The lower row of panels includes all food-web models (Tables S7 to S9). Food-web models designated with a (B) include a latent variable to represent bacteria. The first column of panels includes models with no links to habitat volume (Tables S3, S4, and S7). The second column of panels includes models with limited links to habitat volume (Tables S5 and S8). For the bottom-up food-web models, the link was from habitat volume to prey abundance, and for the top-down food-web models, the link was from habitat volume to the abundances of *Fletcherimyia* and *Wyeomyia*. The third column of panels depicts models with all taxa linked to habitat volume (Tables S6 and S9). The single best-fitting model (*Wyeomyia* keystone with neither partial nor complete links with habitat volume) is indicated with a blue symbol. The group of best-fitting models (food-web models with no volume links) is indicated with red symbols. For reference in each panel, the cross-validation index for the *Wyeomyia* model is indicated by a dashed blue line, and the average cross-validation for the food-web models with no habitat links is indicated by a solid red band.

The overall fit of the set of ecological models to the abundance data was food-web models > single factor models (Figure 2, lower panels versus upper panels). The fit of the food-web and keystone models was not improved by incorporating partial links or complete links with habitat volume (Figure 2, left panel versus center and right panels). The single best-fitting model was the *Wyeomyia* keystone model (blue symbol and dashed line in Figure 2), and the worst-fitting model was the *Sarraceniopus* partial-volume response model. The best-fitting group of models was composed of the four food-web models with no volume linkage (red symbols and red band in Figure 2). These models and the *Wyeomyia* keystone model did not reject the statistical hypothesis of a “close fit” with the variance-covariance structure of the data. Candidate models of other keystone species or of other food-web models with volume linkage performed more poorly.

**Figure 3:**
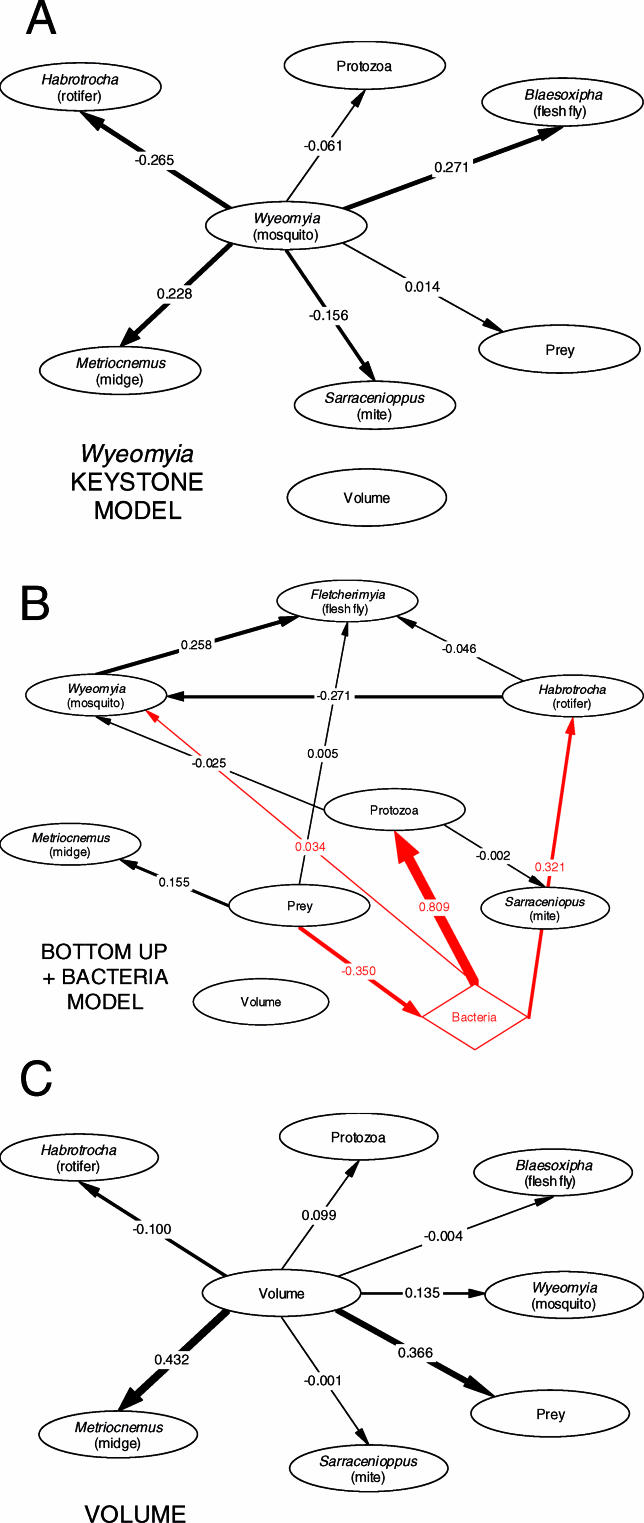
Path Models of *Sarracenia* Food-Web Structure Each circle indicates a taxon abundance, prey abundance, or average habitat volume, and each link indicates a hypothesized cause-and-effect relationship within a particular model. Standardized path coefficients are estimated for each link, and the size of the arrow is roughly proportional to the magnitude of the coefficient. (A) *Wyeomyia* single-factor (“keystone”) model in which abundances of all taxa are determined only by direct links with the filter-feeding mosquito larva W. smithii. (B) Bottom-up trophic model in which abundances are controlled by links from prey to predator. The triangle represents a latent variable for bacteria, which was not directly measured, but whose trophic linkages (shown in red) in the *Sarracenia* web are known [21,24]. (C) Single-factor volume model, in which the abundance of each taxon is determined solely by habitat volume.

The *Wyeomyia* keystone model correctly identified the role of mosquito larvae as predators of rotifers and as prey of *Fletcherimyia* (Figure 3A) [26]. These path analyses are consistent with other studies suggesting that *Wyeomyia* is a keystone predator with both top-down and bottom-up effects in this food web [25,27]. The latent variable analysis of the food-web models suggested that strong trophic links were associated with bacteria (Figure 3B), which supports the view of the *Sarracenia* food web as a commensal processing chain in which shredders, detritivores, and filter feeders sequentially process and transform arthropod prey [23]. Although it fit the data relatively poorly (Figure 2), the simple model linking species abundances only to volume identified a strong link from pitcher-plant volume to prey abundance and a strong link from volume to *Metriocnemus* abundance (Figure 3C). Weaker links were from volume to *Wyeomyia* abundance and *Habrotrocha* abundance, and all other links in this model were close to zero.

## Discussion

The overall superiority of the *Wyeomyia* keystone model and the simple food-web models demonstrates the importance of trophic interactions in controlling species abundances in an aquatic food web. Even though habitat volume was radically altered by our manipulations (Table S1), the abundances of species in the food web were better predicted overall by trophic interaction models than by direct measures of habitat volume or the abundance of basal prey resources alone, or by models linking habitat volume partially or completely to food-web structure (Figure 2). Although a few taxa did show correlations with habitat volume (Figure 3C), the fit of the food-web models to the abundance data was worse when habitat volume linkages were included (Figure 2). Our experimental study confirms the importance of trophic interactions that have been observed on terrestrial islands that have been sharply reduced in area [1] or altered by an invasive species [7]. Explicit statistical models of the trophic structure of communities may provide more accurate forecasts of plant and animal abundances in the face of habitat change than models that focus on the idiosyncratic responses of individual species to habitat loss alone.

## Materials and Methods

At Moose Bog, a peat bog of northeastern Vermont, United States [28], we assigned 50 adult pitcher plants randomly to one of five treatment groups in which we experimentally manipulated the water level of the leaf and/or removed all of the dipteran larvae from the aquatic food web (see Protocol S1). The food web in each of three leaves of the plant underwent census nondestructively once a week through the summer 2000 growing season. We calculated average abundance of each taxon for each food web during the interval that the leaf was open. This averaging eliminated pseudoreplication and autocorrelation of temporal census data. Our analyses thus considered the average conditions that communities achieve in the face of sustained habitat alteration. The experimental treatments significantly altered average invertebrate abundances and generated substantial variation among leaves in habitat volume, prey availability, and assemblage structure (Table S1).

We used path analysis [29] (a form of structural equation modeling) to compare the fit of these abundance data to different models of community organization (see Protocol S1). We developed a priori models of cause-and-effect to potentially account for patterns of covariation in abundance of taxa among leaves that had been manipulated experimentally (Table S2). Two groups of models were developed: (1) single-factor models, in which abundances of each species respond individually to differences in habitat volume (Table S3), prey resources, or the presence of other species (“keystone” models; Tables S4 to S6), and (2) food-web models, in which species abundances respond to direct and indirect trophic interactions (Tables S7 to S9). We did not model the experimental treatments as dummy categorical variables because the habitat volume and dipteran manipulations were not orthogonal and because habitat volume and dipteran abundance are more appropriately treated as continuous variables, along with the other variables in our models. For the food-web models, we also introduced a latent variable to represent the effects of the unmeasured bacterial component of the assemblage.

The single-factor and food-web models were orthogonally crossed with three other groupings, based on the inclusion of habitat volume (= leaf pitcher liquid volume) in the path models. In the first grouping (left pair of panels in Figure 2), habitat volume was not included in the models, so that abundances were predicted entirely on the basis of single-species effects (Table S4) or trophic interactions (Table S7). In the second grouping (middle pair of panels in Figure 2), we introduced habitat volume with only a link to the focal taxon (Tables S5 and S8). In the bottom-up food-web models, this link was from habitat volume to prey, and in the top-down food-web models, the link was from habitat volume to the abundances of *Fletcherimyia* and *Wyeomyia,* the two top predators in this food web (Table S8). In the third grouping (right pair of panels in Figure 2), we modeled links from habitat volume to all food-web components (Tables S6 and S9). All models were fit to the observed correlation matrix for the *Sarracenia* food web (Table S2).

## Supporting Information

Dataset S1 Average Abundances of Inquilines, Average Habitat Volume, Leaf Age, and Treatment (283 KB DOC)

Protocol S1 Experimental Manipulations, Model Structure, and Path Model Analysis (192 KB DOC)

Table S1 Split-Plot Analysis of Variance Results for Tests of Treatment and Leaf Age on Inquiline Abundances (31 KB DOC)

Table S2 Observed Correlation Matrix for Path Analyses (34 KB DOC)

Table S3 Path Structure for Simple Volume Model (34 KB DOC)

Table S4 Path Structure for *Wyeomyia* Keystone Species Model (33 KB DOC)

Table S5 Path Structure for *Wyeomyia* Keystone Species Model with Simple Volume Linkage (33 KB DOC)

Table S6 Path Structure for *Wyeomyia* Keystone Species Model with Full Volume Linkages (33 KB DOC)

Table S7 Path Structure for Top-Down Food-Web Model (33 KB DOC)

Table S8 Path Structure for Top-Down Food-Web Model with Simple Volume Linkage (33 KB DOC)

Table S9 Path Structure for Top-Down Food-Web Model with Full Volume Linkages (34 KB DOC)
