# Supplementary material for: Food-Web Models Predict Species Abundances in Response to Habitat Change
Source: PLoS Biol. 2006 Sep 26;4(10):e324. doi: 10.1371/journal.pbio.0040324 (PMC1570498; doi:10.1371/journal.pbio.0040324)
Supplement: Supplementary file 1 [file pbio.0040324.sd001.doc]

DataSet S1. Complete data set for path analyses.

Treatment: Leaf manipulations: A = Control; B = Trophic Removal; C = Habitat Expansion; D = Habitat Expansion & Trophic Removal; E = Habitat Contraction & Trophic Removal.

Leaf Age: X = Previous year’s leaf; A = First leaf; B = Second leaf.

Plant ID: Unique identification number for each plant assigned to an experimental treatment.

Leaf Volume: Average habitat volume (ml) for each leaf during the experiment.

Protozoa – *Wyeomyia*: Average abundance of individual taxa and prey measured in the experiment. Protozoa data were log_2_(x + 1) transformed.

| **Treatment** | **Plant Age** | **Plant ID** | **Leaf Volume** | **ln(Protozoa)** | **Metriocnemus** | **Fletcherimyia** | **Sarracenioppus** | **Habrotrocha** | **Prey** | **Wyeomyia** |
| --- | --- | --- | --- | --- | --- | --- | --- | --- | --- | --- |
| A | X | A5 | 5.6500 | 0.9316 | 7.0000 | 0.0714 | 0.2308 | 5.1429 | 0.8571 | 0.2857 |
| A | A | A5 | 7.1375 | 4.2084 | 2.6250 | 0.2500 | 0.1250 | 5.8750 | 5.2500 | 4.0000 |
| A | X | A29 | 2.0714 | 3.2728 | 3.0714 | 0.0714 | 0.7692 | 21.3846 | 0.2857 | 0.3571 |
| A | A | A29 | 4.0800 | 0.0000 | 1.8000 | 0.0000 | 0.4000 | 10.0000 | 1.2000 | 4.6000 |
| A | X | A1 | 0.5643 | 2.8861 | 0.2143 | 0.0000 | 0.0000 | 3.1538 | 0.2143 | 0.0000 |
| A | A | A1 | 3.6143 | 2.8983 | 3.1429 | 0.0000 | 0.2857 | 3.4286 | 8.0000 | 3.1429 |
| A | X | A13 | 26.1571 | 2.1812 | 10.7857 | 0.0714 | 0.2857 | 5.7857 | 2.7143 | 3.2857 |
| A | A | A13 | 2.6667 | 2.8717 | 2.0000 | 0.0000 | 0.1667 | 3.5000 | 0.0000 | 3.6667 |
| A | A | A4 | 14.4500 | 2.8717 | 11.1667 | 0.0000 | 1.3333 | 10.3333 | 3.6667 | 1.6667 |
| A | X | A7 | 8.0714 | 0.0000 | 4.2143 | 0.0714 | 0.3846 | 8.3846 | 2.7143 | 0.7143 |
| A | A | A7 | 2.4571 | 3.7809 | 0.8571 | 0.0000 | 0.0000 | 11.8571 | 0.7143 | 0.0000 |
| A | B | A7 | 7.7667 | 4.3307 | 6.2500 | 0.7500 | 0.0000 | 3.2500 | 2.2500 | 1.0000 |
| A | X | A49 | 3.6857 | 8.0943 | 7.4286 | 0.8462 | 0.6154 | 27.0769 | 1.9231 | 0.7143 |
| A | X | A45 | 1.5929 | 3.1813 | 2.2857 | 0.0000 | 0.0769 | 2.1538 | 0.1429 | 0.4286 |
| A | B | A45 | 1.1000 | 0.0000 | 2.0000 | 0.0000 | 0.0000 | 1.0000 | 0.0000 | 7.5000 |
| A | X | A30 | 5.3429 | 2.2472 | 3.3571 | 0.0000 | 0.3077 | 15.1538 | 1.0714 | 0.9286 |
| A | B | A30 | 6.6750 | 0.0000 | 0.7500 | 0.0000 | 2.0000 | 4.0000 | 1.0000 | 1.2500 |
| A | X | A15 | 2.3833 | 1.1451 | 1.3333 | 0.0000 | 0.2143 | 8.6429 | 0.4286 | 0.0000 |
| A | A | A15 | 1.8500 | 0.0000 | 0.7500 | 0.1250 | 0.1250 | 8.1250 | 0.3750 | 0.0000 |
| B | X | B32 | 0.1786 | 1.5782 | 0.4615 | 0.0000 | 0.2308 | 4.6923 | 0.0769 | 0.0000 |
| B | A | B32 | 1.7857 | 3.7809 | 2.5714 | 0.1429 | 0.0000 | 15.4286 | 0.0000 | 0.1429 |
| B | B | B32 | 4.4800 | 3.0445 | 1.2000 | 0.2000 | 1.6000 | 9.6000 | 6.2000 | 0.4000 |
| B | X | B22 | 4.6000 | 0.0000 | 1.0000 | 0.0000 | 2.4692 | 3.0769 | 0.5000 | 0.0714 |
| B | A | B22 | 4.8714 | 5.0173 | 1.8571 | 0.2857 | 0.2857 | 3.2857 | 2.8571 | 0.1429 |
| B | B | B22 | 3.8571 | 4.7499 | 1.4286 | 0.4286 | 1.4286 | 4.8571 | 1.4286 | 0.4286 |
| B | X | B42 | 4.0571 | 0.1542 | 2.8571 | 0.0000 | 0.4167 | 10.2500 | 0.5714 | 1.0769 |
| B | A | B42 | 1.9286 | 2.0971 | 2.1429 | 0.0000 | 0.0000 | 2.4286 | 0.4286 | 0.0000 |
| B | B | B42 | 3.3800 | 0.0000 | 1.4000 | 0.0000 | 0.2000 | 1.4000 | 1.0000 | 1.2000 |
| B | X | B24 | 3.7571 | 2.5883 | 1.0714 | 0.0714 | 0.6923 | 9.2308 | 0.5000 | 0.0000 |
| B | A | B24 | 0.1500 | 0.0000 | 0.6667 | 0.0000 | 0.3333 | 0.3333 | 0.0000 | 1.1667 |
| B | B | B24 | 4.8000 | 4.6151 | 2.0000 | 0.0000 | 0.3333 | 3.6667 | 2.6667 | 12.3333 |
| B | X | B11 | 4.1143 | 2.1624 | 0.8571 | 0.0000 | 0.3077 | 13.3077 | 1.1429 | 0.0000 |
| B | A | B11 | 4.4667 | 3.7136 | 3.0000 | 0.1429 | 0.1429 | 6.4000 | 0.8571 | 1.1429 |
| B | X | B36 | 7.8143 | 2.4323 | 2.6429 | 0.0000 | 0.0000 | 14.1538 | 1.0714 | 0.0000 |
| B | A | B36 | 9.4029 | 3.7809 | 0.4286 | 0.1429 | 0.1429 | 3.7143 | 3.2857 | 0.1429 |
| B | B | B36 | 1.8000 | 0.0000 | 0.5000 | 0.0000 | 0.0000 | 0.5000 | 0.0000 | 13.0000 |
| B | X | B47 | 21.4071 | 0.0000 | 8.0000 | 0.0000 | 0.1538 | 21.5000 | 2.6667 | 2.0000 |
| B | A | B47 | 7.5800 | 2.3979 | 5.2000 | 0.2000 | 2.4000 | 0.4000 | 8.0000 | 0.8000 |
| B | X | B39 | 2.7714 | 0.0000 | 0.6429 | 0.0000 | 0.0769 | 11.5000 | 0.5000 | 0.0714 |
| B | A | B39 | 4.2600 | 0.0000 | 5.0000 | 0.2000 | 0.4000 | 1.2000 | 4.0000 | 1.2000 |
| B | X | B37 | 10.4929 | 2.1624 | 6.0714 | 0.0000 | 0.0769 | 17.5385 | 0.9286 | 1.0000 |
| B | A | B37 | 4.9286 | 7.1374 | 0.4286 | 0.2857 | 0.7143 | 14.0000 | 1.5714 | 0.8571 |
| C | X | C50 | 13.2571 | 4.5523 | 8.9286 | 0.0000 | 0.2143 | 18.6154 | 1.0769 | 0.3541 |
| C | A | C50 | 7.0375 | 0.8109 | 1.8750 | 0.1250 | 0.0000 | 7.0000 | 0.5000 | 0.0000 |
| C | B | C50 | 23.5000 | 3.9318 | 1.3333 | 0.3333 | 0.0000 | 5.5000 | 2.1667 | 1.6667 |
| C | X | C16 | 2.1714 | 0.0000 | 0.7143 | 0.0000 | 0.6923 | 11.2308 | 0.5000 | 0.0000 |
| C | A | C16 | 1.0500 | 0.0000 | 0.2000 | 0.0000 | 0.0000 | 0.4000 | 0.0000 | 0.0000 |
| C | X | C35 | 4.6429 | 1.7255 | 0.5714 | 0.0000 | 0.4615 | 17.3846 | 0.2143 | 0.0000 |
| C | A | C35 | 4.8333 | 0.0000 | 0.8333 | 0.0000 | 0.1667 | 1.6667 | 0.0000 | 0.0000 |
| C | X | C34 | 12.0429 | 1.5782 | 1.6154 | 0.0000 | 0.6154 | 9.1538 | 0.7857 | 1.2143 |
| C | B | C34 | 19.9000 | 1.4663 | 4.3333 | 0.0000 | 0.8333 | 2.1667 | 7.5000 | 0.8333 |
| C | X | C12 | 20.4000 | 0.9316 | 5.9286 | 0.0000 | 0.3333 | 11.4615 | 1.2143 | 0.0000 |
| C | A | C12 | 4.9714 | 1.3499 | 3.0000 | 0.1429 | 0.1429 | 4.1429 | 14.5714 | 0.4286 |
| C | B | C12 | 43.5000 | 3.4340 | 0.2500 | 0.2500 | 0.5000 | 2.5000 | 9.7500 | 1.2500 |
| C | X | C17 | 36.3429 | 0.0000 | 1.2143 | 0.0000 | 0.4615 | 2.4615 | 1.2857 | 0.9286 |
| C | A | C17 | 15.8333 | 3.2581 | 1.3333 | 0.1667 | 0.3333 | 0.8333 | 5.8333 | 2.6667 |
| C | X | C14 | 10.8357 | 0.3254 | 2.2143 | 0.0000 | 1.1538 | 12.7692 | 1.0000 | 0.3077 |
| C | A | C14 | 13.9000 | 2.8717 | 3.0000 | 0.6667 | 0.0000 | 1.0000 | 2.0000 | 2.0000 |
| C | X | C44 | 4.3786 | 2.1624 | 1.9286 | 0.0000 | 0.1538 | 5.8462 | 0.2143 | 0.7143 |
| C | A | C44 | 21.2833 | 1.7636 | 13.0000 | 0.1667 | 0.1667 | 0.1667 | 0.0000 | 3.6667 |
| C | X | C6 | 5.0500 | 1.7255 | 0.5000 | 0.7143 | 0.1538 | 4.4615 | 3.8571 | 0.0000 |
| C | A | C6 | 25.2000 | 3.9318 | 1.5000 | 0.3750 | 0.2500 | 1.8750 | 8.6250 | 1.0000 |
| C | B | C6 | 18.4200 | 3.4340 | 2.0000 | 0.0000 | 0.0000 | 2.6000 | 6.6000 | 0.5000 |
| C | X | C8 | 7.1143 | 2.2057 | 2.9286 | 0.0000 | 0.3846 | 24.2308 | 1.2143 | 0.6429 |
| C | A | C8 | 26.2143 | 3.9318 | 1.1429 | 0.2857 | 0.0000 | 2.1429 | 5.2857 | 0.1429 |
| C | B | C8 | 17.0000 | 3.8067 | 0.8000 | 0.2000 | 0.0000 | 2.6000 | 2.8000 | 1.8000 |
| D | X | D3 | 4.6429 | 0.0000 | 3.2857 | 0.0000 | 0.6923 | 4.6154 | 0.3571 | 1.3571 |
| D | A | D3 | 5.8143 | 0.0000 | 1.0000 | 0.0000 | 0.2857 | 5.2857 | 9.1667 | 1.2857 |
| D | B | D3 | 14.1500 | 4.0832 | 4.0000 | 0.6667 | 0.6667 | 5.8333 | 5.5000 | 1.5000 |
| D | X | D46 | 12.9714 | 3.4942 | 9.7857 | 0.0000 | 0.3077 | 5.3077 | 1.2143 | 3.4286 |
| D | A | D46 | 10.1571 | 4.2826 | 6.1429 | 0.0000 | 0.1429 | 4.4286 | 2.0000 | 5.5714 |
| D | B | D46 | 3.7500 | 0.0000 | 8.7500 | 4.2500 | 0.0000 | 0.0000 | 0.0000 | 10.1500 |
| D | X | D43 | 7.8000 | 5.7424 | 6.6000 | 0.0000 | 0.2000 | 13.5000 | 0.9000 | 1.4000 |
| D | A | D43 | 19.0375 | 2.7726 | 7.1250 | 0.0000 | 0.1250 | 9.8750 | 5.7500 | 2.0000 |
| D | B | D43 | 27.6000 | 0.0000 | 3.4000 | 0.2000 | 0.8000 | 6.4000 | 2.6000 | 6.4000 |
| D | X | D33 | 50.5512 | 3.2432 | 18.2857 | 0.0000 | 0.3846 | 3.5385 | 2.5000 | 3.8571 |
| D | A | D33 | 34.6400 | 0.0000 | 1.6000 | 0.2000 | 0.2500 | 1.5000 | 2.0000 | 4.2000 |
| D | X | D40 | 23.1786 | 0.6506 | 16.9286 | 0.0000 | 0.0000 | 3.7500 | 2.4286 | 2.6429 |
| D | A | D40 | 21.0714 | 4.3021 | 16.1429 | 0.0000 | 0.0000 | 3.2857 | 8.2857 | 3.7143 |
| D | B | D40 | 2.5400 | 1.0986 | 2.2000 | 0.0000 | 0.0000 | 1.4000 | 0.2000 | 3.8000 |
| D | X | D31 | 9.6071 | 0.5705 | 2.3571 | 0.0000 | 0.7692 | 15.3846 | 1.7692 | 0.2857 |
| D | A | D31 | 18.1000 | 2.3979 | 1.2857 | 0.1429 | 0.0000 | 7.1429 | 2.8571 | 2.2857 |
| D | B | D31 | 3.3333 | 0.0000 | 0.6667 | 0.0000 | 0.0000 | 6.0000 | 0.3333 | 7.6667 |
| D | X | D10 | 7.5143 | 0.5705 | 1.0714 | 0.0714 | 0.1538 | 20.6154 | 0.7143 | 0.6429 |
| D | A | D10 | 18.5625 | 2.2773 | 6.3750 | 0.0000 | 0.6250 | 7.1250 | 28.5000 | 2.1250 |
| D | X | D9 | 4.3500 | 2.5883 | 2.5000 | 0.0000 | 0.3077 | 26.4615 | 1.0769 | 0.1429 |
| D | A | D9 | 9.1625 | 2.1401 | 2.1250 | 0.6250 | 0.8750 | 5.2500 | 5.2857 | 0.8750 |
| D | B | D9 | 9.7667 | 0.0000 | 1.0000 | 0.3333 | 0.3333 | 6.6667 | 2.3333 | 0.3333 |
| D | X | D41 | 9.2571 | 2.3191 | 3.4286 | 0.0000 | 2.1538 | 8.7500 | 0.2857 | 0.3571 |
| D | A | D41 | 8.0000 | 0.0000 | 2.1429 | 0.0000 | 0.5714 | 1.4286 | 0.1429 | 0.2857 |
| D | B | D41 | 15.4500 | 0.0000 | 1.6667 | 0.1667 | 0.6667 | 2.6667 | 1.6667 | 2.1667 |
| D | X | D19 | 7.9857 | 1.4053 | 2.5714 | 0.0000 | 0.4615 | 7.0769 | 0.8462 | 0.9286 |
| D | A | D19 |  | 1.8083 | 2.7262 | 0.1406 | 0.7493 | 8.1782 | 5.0172 | 0.9018 |
| E | X | E25 | 1.8071 | 0.9316 | 1.0000 | 0.0000 | 0.0000 | 1.0769 | 0.0000 | 0.6429 |
| E | A | E25 | 2.0000 | 4.6151 | 14.2500 | 0.0000 | 0.2500 | 1.7500 | 0.5000 | 4.2500 |
| E | X | E38 | 2.4286 | 0.5705 | 1.4286 | 0.0000 | 0.0000 | 9.8462 | 0.0714 | 0.5000 |
| E | A | E38 | 0.7600 | 0.0000 | 0.6000 | 0.2000 | 0.0000 | 1.0000 | 1.0000 | 2.6000 |
| E | X | E21 | 0.0929 | 0.0000 | 0.0000 | 0.0000 | 0.0714 | 3.0000 | 0.0000 | 0.0000 |
| E | A | E21 | 2.0000 | 5.9655 | 2.1250 | 0.0000 | 0.0000 | 5.7500 | 0.1250 | 0.1250 |
| E | X | E18 | 2.3857 | 0.0000 | 0.8571 | 0.0000 | 0.0769 | 1.3077 | 1.1429 | 0.0000 |
| E | A | E18 | 2.1000 | 4.0832 | 1.3333 | 0.1667 | 1.0000 | 13.3333 | 1.8333 | 0.0000 |
| E | X | E2 | 5.0643 | 3.2127 | 2.7857 | 0.0000 | 0.6154 | 13.0000 | 0.7143 | 0.2857 |
| E | A | E2 | 1.2333 | 2.8717 | 1.6667 | 0.0000 | 0.3333 | 11.1667 | 0.8333 | 0.8333 |
| E | B | E2 | 1.2667 | 0.0000 | 0.6667 | 0.0000 | 0.0000 | 4.3333 | 0.3333 | 3.6667 |
| E | X | E27 | 1.8143 | 0.0000 | 0.7143 | 0.0000 | 0.0000 | 0.8462 | 0.0000 | 0.2143 |
| E | A | E27 | 0.4857 | 0.0000 | 3.3333 | 0.0000 | 2.7143 | 1.2857 | 0.4286 | 0.0000 |
| E | B | E27 | 0.6000 | 3.5361 | 0.0000 | 0.0000 | 1.0000 | 4.6667 | 0.6667 | 1.3333 |
| E | X | E48 | 3.0857 | 0.0000 | 5.1429 | 0.0000 | 0.0769 | 6.3077 | 0.0714 | 0.2143 |
| E | A | E48 | 1.9800 | 0.0000 | 1.6000 | 0.0000 | 0.0000 | 6.4000 | 1.2000 | 0.2000 |
| E | B | E48 | 2.5000 | 0.0000 | 1.5000 | 0.2500 | 0.0000 | 5.7500 | 0.2500 | 2.7500 |
| E | X | E23 | 1.6714 | 1.7255 | 1.7143 | 0.0000 | 0.0000 | 3.4615 | 0.2143 | 0.9286 |
| E | A | E23 | 3.7125 | 3.9318 | 4.7500 | 0.3750 | 0.3750 | 6.2500 | 2.2500 | 0.0000 |
| E | B | E23 | 3.0143 | 4.7474 | 2.4286 | 0.1429 | 1.1429 | 6.1429 | 1.0000 | 0.7143 |
| E | X | E26 | 1.3714 | 0.0000 | 2.2143 | 0.5714 | 0.0000 | 4.7143 | 0.3571 | 0.1429 |
| E | A | E26 | 4.5000 | 0.9808 | 0.5000 | 0.0000 | 1.0000 | 10.3333 | 0.1667 | 2.6667 |
| E | B | E26 | 1.0000 | 0.0000 | 1.7500 | 0.2500 | 0.0000 | 11.0000 | 0.2500 | 1.5000 |
| E | X | E28 | 1.7071 | 0.0000 | 0.2857 | 0.0000 | 0.3077 | 5.4615 | 0.0714 | 0.7143 |
| E | A | E28 | 1.3833 | 3.7136 | 2.0000 | 0.3333 | 0.0000 | 6.8000 | 2.1667 | 0.1667 |
| E | B | E28 | 1.0250 | 3.9318 | 0.5000 | 0.2500 | 0.0000 | 6.2500 | 1.2500 | 0.2500 |
