# Supplementary material for: Food-Web Models Predict Species Abundances in Response to Habitat Change
Source: PLoS Biol. 2006 Sep 26;4(10):e324. doi: 10.1371/journal.pbio.0040324 (PMC1570498; doi:10.1371/journal.pbio.0040324)
Supplement: Supplementary file 2 [file pbio.0040324.sd002.doc]

Protocol S1

Experimental Manipulations

The study was conducted at Moose Bog, an 86-ha peatland in northeastern Vermont, USA [1]. Fifty non-flowering pitcher plants with rosette diameters > 8 cm were haphazardly chosen at the start of the study (15 May 2000). All plants were located in the center of the *Sphagnum* mat, in full sun, at least 0.75 m from the nearest neighbor. Plants were assigned randomly to one of 5 experimental treatments:

1) Control. Diptera larvae and pitcher liquid were removed and censused, and then returned to leaf.

2) Trophic removal. All diptera larvae and pitcher liquid were removed and censused, and the leaf was refilled with an equal volume of distilled water.

3) Habitat expansion. All diptera larvae and pitcher liquid were removed and censused, and then returned to the leaf. The leaf was then topped up with to the brim with additional distilled water as needed.

4) Habitat expansion & trophic removal. All diptera larvae and pitcher liquid were removed and censused. The leaf was then filled to the brim with distilled water.

5) Habitat contraction & trophic removal. All diptera larvae and pitcher liquid were removed and censused.

These treatments mimicked changes in habitat volume (treatments 3, 4, and 5) and removal of top trophic levels from the food web (treatments 2, 4, and 5). Changes in habitat volume also affect the food chain base, because average *Sarracenia* prey capture was highest in treatments 3 and 4 (habitat expansion) and lowest in treatment 5 (habitat contraction). Because the liquid in a pitcher plant cannot be completely removed, the trophic removal and habitat contraction treatments probably do not eliminate populations of basal fauna and flora (bacteria, protozoa, rotifers, mites, algae, and yeast, all of which can recolonize rapidly), but they do remove the large dipteran larvae that shred the prey (*Metriocnemus knabi* and small *Fletcherimyia fletcheri*) or constitute the upper trophic levels (*Wyeomyia smithii* and large *F. fletcheri*). However, the requirement of the top predators *F. fletcheri and W. smithii* for pools of standing water meant that it was impossible to add a sixth treatment – habitat contraction without trophic removal – that would have given us a complete two-factor design.

Treatments were applied twice weekly from 1 May 2000 through 15 August 2000 to all the open leaves of the plant. Because the summer of 2000 was cool and rainy, two treatment applications per week were necessary to maintain differences among treatments in water volume of the leaves. Even with two treatment applications per week, most leaves in the habitat contraction & trophic removal treatment still contained small quantities of pitcher liquid and an associated animal community. Average habitat volumes (mean ± SE of pitcher liquid) in the 5 treatments were: Control (5.6 ml ± 1.78); trophic removal (5.65 ml ± 1.62); habitat expansion (14.95 ml ± 1.58); habitat expansion & trophic removal (13.80 ml ± 1.62); habitat contraction & trophic removal (1.96 ml ± 1.52).

Three leaves on each plant were marked and censused from 31 May to 23 August 2000: A) The largest intact mature leaf from the 1999 growing season; B) The first leaf to open in the 2000 growing season; C) The second leaf to open in the 2000 growing season. Some leaves were damaged by herbivorous moth (*Exyra fax*) larvae or vertebrates; damaged leaves that could not hold water through the season were not included in the analysis. In addition, 20 plants did not produce a second leaf, and one plant died during the experiment. Data were obtained from 118 out of 150 possible leaves (5 treatments × 3 leaf ages × 10 replicates); missing observations were distributed randomly among treatments (DataSet S1). The time-scale of this experiment, which lasted for the majority of a single field season, is appropriate for determining average responses of the *Sarracenia* food web. The *Sarracenia* food web assembles within a few days to weeks after a leaf opens [2]. Moreover, the major taxa that are associated with *Sarracenia* are fairly common, and their identities do not vary geographically [3]. Thus, it is unlikely that transient taxa excluded by predators might show up in a more extended experiment and alter our findings.

Macroinvertebrates were censused weekly immediately before the second treatment application by carefully suctioning the contents of each leaf with a 50 ml plastic syringe and a short length of plastic tubing. The volume of the pitcher liquid (= habitat volume) was recorded, and the liquid was emptied into a plastic petri dish. The number of invertebrate prey head capsules and the number of living dipteran larvae (*Metriocnemus*, *Fletcherimyia*, and *Wyeomyia*) were counted in the field under a hand lens. A 0.5 ml subsample of pitcher liquid was collected from each leaf and stored in a labeled microcentrifuge tube. Tubes were refrigerated and mailed overnight to L. Bledzski (Mt. Holyoke College, S. Hadley, MA, USA), who provided microscope counts of total protozoa, *Sarraceniopus*, and *Habrotrocha*. Protozoa were not identified to species, and bacteria, algae, and yeast were not censused. Pitcher liquid, invertebrates, and prey were either replaced in the leaf or discarded, depending on the treatment. Between each leaf census, the petri dish, syringe, and plastic tubing were rinsed with distilled water to prevent cross-contamination.

For each leaf, we first calculated the average habitat volume, and the average abundance of prey, *Fletcherimyia*, *Metriocnemus*, *Wyeomyia*, *Habrotrocha*, *Sarraceniopus*, and protozoa for all those censuses in which the leaf was open. Analysis of the average values for each leaf allowed for statistical comparison of all of the leaves (which open at different times during the growing season), reduced variation due to sampling error, and avoided pseudoreplication of weekly census numbers (i.e., incorrectly treating weekly censuses as independent replicates). We included all zeroes in these averages, as they represent real zeroes: observations in which taxa were not observed [4]. Although macroinvertebrates may be present and dormant or undetectable in low-volume pitchers, they are not biologically active, and it would be misleading to use data only from censuses for which taxa were present. Including zeroes biases the path analyses in favor of the single-factor models, because zeroes were often associated with low pitcher volumes. *Wyeomyia* pupae and larvea were summed, and protozoa counts were log transformed; otherwise, average abundances of taxa for each leaf were not statistically transformed prior to analysis.

Model Structure

Community ecologists usually fit the relative abundance of species in an assemblage to niche-based resource partitioning models [5], and more recently to neutral models [6]. However, both the niche based models and the neutral model are typically restricted to the analysis of species at the same trophic or feeding level. We developed a series of *a priori* path models to depict different mechanistic hypotheses for the control of abundance in a trophic web:

*Single Factor Models.* In these models, the abundance of each taxon is controlled by only a single controlling variable. This class of models includes the most basic model in which abundance is controlled solely by habitat volume (Table S3). This class of models also includes single-species and keystone-species effects (Table S4), in which each of the 6 major taxa (*Sarraceniopus*, *Fletcherimyia*, Protozoa, *Metriocnemus*, *Habrotrocha*, and *Wyeomyia*) are analyzed as a putative keystone species. In the keystone species model, abundances of all other taxa are directly linked to the keystone species, with no indirect interactions included.

*Food-web models.* Four path models in which links between taxa (including prey) represent known predator-prey relationships in the *Sarracenia* food web (Table S7). In the bottom-up models, all links are from prey to predator. In the top-down models, all links are from predator to prey. Because the direction of the links had negligible effects on model fitting, the estimated coefficients, statistical significance, and cross-validation index were virtually identical for the top-down and bottom-up models. For both the top-down and bottom-up models, we first fit models that included only links between macroinvertebrates for which we had directly measured abundance. We then fit models that included a latent variable to represent bacteria, and incorporated known trophic links from prey to bacteria, bacteria to protozoa, and bacteria to *Habrotrocha*.

The single-factor and food-web models were orthogonally crossed with an additional set of three groupings:

*No volume links.* In these models, habitat volume was entirely excluded, and the models included only the effects of the interacting taxa.

*Partial volume links.* In these models (Tables S5, S8), habitat volume was linked only to the focal taxon in the model. For the bottom-up food web models, the link was from habitat volume to prey abundance, whereas for the top-down food web models, the link was from habitat volume to the abundances of *Fletcherimyia* and *Wyeomyia*.

*Full volume links.* In these models, habitat volume was linked to all tax in the model (Tables S6, S9).

The correlation matrix for the observed data is given in Table S2, and the variance-covariance structures for the simple habitat volume model and for a representative model from each of the 6 groups is given in Tables S3-S9.

Path Model Analysis

In the path analyses (a form of structural equation modeling), each individual leaf is treated as an independent replicate. This assumption is justified because ANOVAs indicated negligible variation in macroinvertebrate abundance among plants within a treatment. For the path analyses, we deliberately pooled all replicates across the 5 experimental treatments. The rationale for pooling is that the treatments create different patterns of abundance and community structure, and the path analysis tries to account for that variation with different cause-and-effect models, which are expressed as simple path diagrams. All path models were fit to the same correlation matrix based on complete data set of censused leaves (Table S2).

We used maximum likelihood methods in Systat 10.0 to calculate standardized path coefficients and their 90% confidence intervals. To assess the relative fit of the data to each model, we calculated an expected cross validation index (ECVI) and 90% confidence inteval. The point estimate of the ECVI is related by a linear transformation to Akaike’s Information Criterion [7]. Thus, the smaller the index, the better fit of the model to the data, adjusted for the number of parameters. For each model, we calculated the exceedance probability for a test of the null hypothesis of a "close fit" of the data to the model covariance structure. We have not presented the statistical significance of the standardized path coefficients of each model, nor have we placed much emphasis on the relative sizes of the coefficients, which may not always be appropriate for ecological inferences [8]. Instead, we have emphasized the overall fit of the data to each path model, which represents an *a priori* hypothesis about the control of abundance.

Supplemental References

1. Gotelli NJ, Ellison, AM (2002) Biogeography at a regional scale: determinants of ant species density in bogs and forests of New England. Ecology 83:1604-1609.

2. Ellison AM, Gotelli NJ, Brewer JS, Cochran-Stafira DL, Kneitel J, et al. (2003) The evolutionary ecology of carnivorous plants. Adv Ecol Res 33:1-74.

3. Buckley HL, Miller TE, Ellison AM, Gotelli NJ (2003) Reverse latitudinal trends in species richness of pitcher-plant food webs. Ecol Lett 6:825-829.

4. Martin TG, Wintle BA, Rhodes JR, Kuhnert PM, Field SA, et al. (2005) Zero tolerance ecology: improving ecological inference by modelling the source of zero observations. Ecol Lett 8:1235-1246.

5. Hubbell, SP (2001) The unified neutral theory of biodiversity and biogeography. Princeton: Princeton University Press.

6. Chase JM, Leibold MA. (2003) Ecological niches: Linking classical and contemporary approaches. Chicago: University of Chicago Press.

7. Burnham KP, Anderson DR (2002) Model selection and inference: A practical information-theoretic approach. New York: Springer-Verlag.

8. Grace JB, Bollen KA (2005) Interpreting the results from multiple regression and structural equation models. Bull Ecol Soc Am 86:283-295.

Table S1. Results of split-plot ANOVAs to test for effects of experimental treatment and leaf age. Habitat Volume = volume of pitcher liquid. Prey = number of arthropod head capsules counted in each leaf. A principal components analysis was conducted on the abundances all of the invertebrate taxa (*Fletcherimyia*, *Wyeomyia*, *Habrotrocha*, *Metriocnemus*, *Sarraceniopus*) and protozoa to create a single score that quantified overall patterns of abundance in the assemblage. The first two principal components (PC1 and PC2) captured 86% of the variation in abundance. For PC1, the largest factor loadings were for *Habrotrocha* (0.989) and *Wyeomyia* (-0.118). For PC2, the largest factor loadings were for *Metriocnemus* (-0.960) and *Wyeomyia* (-0.225). Each row lists a different response variable in the split-plot ANOVA. Each column gives the different statistical tests, with the numerator and denominator degrees of freedom listed in parentheses. The entries in the table are the *P*-values for each F-ratio. Bold-face indicates statistically significant effects (*P* < 0.05).

| **Response Variable** | **Treatment (4, 36)** | **Leaf Age (2, 59)** | **T x A (8, 59)** |
| --- | --- | --- | --- |
| Habitat Volume | **<0.001** | 0.516 | 0.272 |
| Prey | **0.018** | **< 0.001** | 0.214 |
| PC1 | 0.830 | **< 0.001** | 0.214 |
| PC2 | **0.043** | 0.704 | 0.445 |
| *Fletcherimyia* | 0.633 | **0.045** | 0.346 |
| *Wyeomyia* | 0.059 | **<0.001** | 0.100 |
| *Habrotrocha* | 0.799 | **<0.001** | **0.007** |
| *Metriocnemus* | **0.047** | 0.207 | 0.444 |
| *Sarraceniopus* | 0.716 | 0.957 | 0.410 |
| Protozoa | 0.703 | 0.057 | 0.241 |

Table S2. Observed correlation matrix for *Sarracenia* food web. Vol = habitat volume; Prey = prey abundance; Proto = protozoa abundance; Fletch = *Fletcherimyia* (flesh fly larva) abundance; Wyeo = *Wyeomyia* (mosquito larva) abundance; Metrio = *Metriocnemus* (midge larva) abundance; Habro = *Habrotrocha* (rotifer) abundance; Sarra = *Sarraceniopus* (mite) abundance.

|  | Vol | Prey | Proto | Fletch | Wyeo | Metrio | Habro | Sarra |
| --- | --- | --- | --- | --- | --- | --- | --- | --- |
| Vol | 1.000 |  |  |  |  |  |  |  |
| Prey | 0.366 | 1.000 |  |  |  |  |  |  |
| Proto | 0.099 | 0.177 | 1.000 |  |  |  |  |  |
| Fletch | -0.004 | 0.014 | 0.061 | 1.000 |  |  |  |  |
| Wyeo | 0.135 | 0.016 | -0.061 | 0.271 | 1.000 |  |  |  |
| Metrio | 0.432 | 0.156 | 0.211 | 0.113 | 0.228 | 1.000 |  |  |
| Habro | -0.100 | -0.114 | 0.218 | -0.115 | -0.264 | 0.033 | 1.000 |  |
| Sarra | -0.001 | 0.090 | -0.001 | -0.067 | -0.154 | -0.020 | 0.046 | 1.000 |

Table S3. Path structure for simple volume model. + = causal link between two variables in the model. 0 = no causal link. s(x) = variance in (x). Columns indicate causal variables and rows indicate response variables. Variable abbreviations as in Table S2.

|  | Vol | Prey | Proto | Fletch | Wyeo | Metrio | Habro | Sarra |
| --- | --- | --- | --- | --- | --- | --- | --- | --- |
| Vol | s(Vol) | 0 | 0 | 0 | 0 | 0 | 0 | 0 |
| Prey | + | s(Prey) | 0 | 0 | 0 | 0 | 0 | 0 |
| Proto | + | 0 | s(Proto) | 0 | 0 | 0 | 0 | 0 |
| Fletch | + | 0 | 0 | s(Fletch) | 0 | 0 | 0 | 0 |
| Wyeo | + | 0 | 0 | 0 | s(Wyeo) | 0 | 0 | 0 |
| Metrio | + | 0 | 0 | 0 | 0 | s(Metrio) | 0 | 0 |
| Habro | + | 0 | 0 | 0 | 0 | 0 | s(Habro) | 0 |
| Sarra | + | 0 | 0 | 0 | 0 | 0 | 0 | s(Sarra) |

Table S4 Path structure for the single-factor *Wyeomyia* keystone species model. Symbols and abbreviations as in Table S3.

|  | Vol | Prey | Proto | Fletch | Wyeo | Metrio | Habro | Sarra |
| --- | --- | --- | --- | --- | --- | --- | --- | --- |
| Vol | 0 | 0 | 0 | 0 | 0 | 0 | 0 | 0 |
| Prey | 0 | s(Prey) | 0 | 0 | + | 0 | 0 | 0 |
| Proto | 0 | 0 | s(Proto) | 0 | + | 0 | 0 | 0 |
| Fletch | 0 | 0 | 0 | s(Fletch) | + | 0 | 0 | 0 |
| Wyeo | 0 | 0 | 0 | 0 | s(Wyeo) | 0 | 0 | 0 |
| Metrio | 0 | 0 | 0 | 0 | + | s(Metrio) | 0 | 0 |
| Habro | 0 | 0 | 0 | 0 | + | 0 | s(Habro) | 0 |
| Sarra | 0 | 0 | 0 | 0 | + | 0 | 0 | s(Sarra) |

Table S5. Path structure for *Wyeomyia* keystone species + limited volume model. Symbols and abbreviations as in Table S3.

|  | Vol | Prey | Proto | Fletch | Wyeo | Metrio | Habro | Sarra |
| --- | --- | --- | --- | --- | --- | --- | --- | --- |
| Vol | s(Vol) | 0 | 0 | 0 | 0 | 0 | 0 | 0 |
| Prey | 0 | s(Prey) | 0 | 0 | + | 0 | 0 | 0 |
| Proto | 0 | 0 | s(Proto) | 0 | + | 0 | 0 | 0 |
| Fletch | 0 | 0 | 0 | s(Fletch) | + | 0 | 0 | 0 |
| Wyeo | + | 0 | 0 | 0 | s(Wyeo) | 0 | 0 | 0 |
| Metrio | 0 | 0 | 0 | 0 | + | s(Metrio) | 0 | 0 |
| Habro | 0 | 0 | 0 | 0 | + | 0 | s(Habro) | 0 |
| Sarra | 0 | 0 | 0 | 0 | + | 0 | 0 | s(Sarra) |

Table S6. Path structure for *Wyeomyia* keystone species + full volume model. Symbols and abbreviations as in Table S3.

|  | Vol | Prey | Proto | Fletch | Wyeo | Metrio | Habro | Sarra |
| --- | --- | --- | --- | --- | --- | --- | --- | --- |
| Vol | s(Vol) | 0 | 0 | 0 | 0 | 0 | 0 | 0 |
| Prey | + | s(Prey) | 0 | 0 | + | 0 | 0 | 0 |
| Proto | + | 0 | s(Proto) | 0 | + | 0 | 0 | 0 |
| Fletch | + | 0 | 0 | s(Fletch) | + | 0 | 0 | 0 |
| Wyeo | + | 0 | 0 | 0 | s(Wyeo) | 0 | 0 | 0 |
| Metrio | + | 0 | 0 | 0 | + | s(Metrio) | 0 | 0 |
| Habro | + | 0 | 0 | 0 | + | 0 | s(Habro) | 0 |
| Sarra | + | 0 | 0 | 0 | + | 0 | 0 | s(Sarra) |

Table S7. Path structure for top-down model. Symbols and abbreviations as in Table S3.

|  | Vol | Prey | Proto | Fletch | Wyeo | Metrio | Habro | Sarra |
| --- | --- | --- | --- | --- | --- | --- | --- | --- |
| Vol | 0 | 0 | 0 | 0 | 0 | 0 | 0 | 0 |
| Prey | 0 | s(Prey) | + | + | 0 | + | 0 | 0 |
| Proto | 0 | 0 | s(Proto) | 0 | + | 0 | 0 | + |
| Fletch | 0 | 0 | 0 | s(Fletch) | 0 | 0 | 0 | 0 |
| Wyeo | 0 | 0 | 0 | + | s(Wyeo) | 0 | 0 | 0 |
| Metrio | 0 | 0 | 0 | 0 | 0 | s(Metrio) | 0 | 0 |
| Habro | 0 | 0 | 0 | + | + | 0 | s(Habro) | 0 |
| Sarra | 0 | 0 | 0 | 0 | 0 | 0 | 0 | s(Sarra) |

Table S8. Path structure for top-down + limited volume model. Symbols and abbreviations as in Table S3.

|  | Vol | Prey | Proto | Fletch | Wyeo | Metrio | Habro | Sarra |
| --- | --- | --- | --- | --- | --- | --- | --- | --- |
| Vol | s(Vol) | 0 | 0 | 0 | 0 | 0 | 0 | 0 |
| Prey | 0 | s(Prey) | + | + | 0 | + | 0 | 0 |
| Proto | 0 | 0 | s(Proto) | 0 | + | 0 | 0 | + |
| Fletch | + | 0 | 0 | s(Fletch) | 0 | 0 | 0 | 0 |
| Wyeo | + | 0 | 0 | + | s(Wyeo) | 0 | 0 | 0 |
| Metrio | 0 | 0 | 0 | 0 | 0 | s(Metrio) | 0 | 0 |
| Habro | 0 | 0 | 0 | + | + | 0 | s(Habro) | 0 |
| Sarra | 0 | 0 | 0 | 0 | 0 | 0 | 0 | s(Sarra) |

Table S9. Path structure for top-down + full volume model. Symbols and abbreviations as in Table S3.

|  | Vol | Prey | Proto | Fletch | Wyeo | Metrio | Habro | Sarra |
| --- | --- | --- | --- | --- | --- | --- | --- | --- |
| Vol | s(Vol) | 0 | 0 | 0 | 0 | 0 | 0 | 0 |
| Prey | + | s(Prey) | + | + | 0 | + | 0 | 0 |
| Proto | + | 0 | s(Proto) | 0 | + | 0 | 0 | + |
| Fletch | + | 0 | 0 | s(Fletch) | 0 | 0 | 0 | 0 |
| Wyeo | + | 0 | 0 | + | s(Wyeo) | 0 | 0 | 0 |
| Metrio | + | 0 | 0 | 0 | 0 | s(Metrio) | 0 | 0 |
| Habro | + | 0 | 0 | + | + | 0 | s(Habro) | 0 |
| Sarra | + | 0 | 0 | 0 | 0 | 0 | 0 | s(Sarra) |
