# Supplementary material for: Food-Web Models Predict Species Abundances in Response to Habitat Change
Source: PLoS Biol. 2006 Sep 26;4(10):e324. doi: 10.1371/journal.pbio.0040324 (PMC1570498; doi:10.1371/journal.pbio.0040324)
Supplement: Supplementary file 3 [file pbio.0040324.st001.doc]

Table S1. Results of split-plot ANOVAs to test for effects of experimental treatment and leaf age. Habitat Volume = volume of pitcher liquid. Prey = number of arthropod head capsules counted in each leaf. A principal components analysis was conducted on the abundances all of the invertebrate taxa (*Fletcherimyia*, *Wyeomyia*, *Habrotrocha*, *Metriocnemus*, *Sarraceniopus*) and protozoa to create a single score that quantified overall patterns of abundance in the assemblage. The first two principal components (PC1 and PC2) captured 86% of the variation in abundance. For PC1, the largest factor loadings were for *Habrotrocha* (0.989) and *Wyeomyia* (-0.118). For PC2, the largest factor loadings were for *Metriocnemus* (-0.960) and *Wyeomyia* (-0.225). Each row lists a different response variable in the split-plot ANOVA. Each column gives the different statistical tests, with the numerator and denominator degrees of freedom listed in parentheses. The entries in the table are the *P*-values for each F-ratio. Bold-face indicates statistically significant effects (*P* < 0.05).

| **Response Variable** | **Treatment (4, 36)** | **Leaf Age (2, 59)** | **T x A (8, 59)** |
| --- | --- | --- | --- |
| Habitat Volume | **<0.001** | 0.516 | 0.272 |
| Prey | **0.018** | **< 0.001** | 0.214 |
| PC1 | 0.830 | **< 0.001** | 0.214 |
| PC2 | **0.043** | 0.704 | 0.445 |
| *Fletcherimyia* | 0.633 | **0.045** | 0.346 |
| *Wyeomyia* | 0.059 | **<0.001** | 0.100 |
| *Habrotrocha* | 0.799 | **<0.001** | **0.007** |
| *Metriocnemus* | **0.047** | 0.207 | 0.444 |
| *Sarraceniopus* | 0.716 | 0.957 | 0.410 |
| Protozoa | 0.703 | 0.057 | 0.241 |
