# Supplementary material for: Food-Web Models Predict Species Abundances in Response to Habitat Change
Source: PLoS Biol. 2006 Sep 26;4(10):e324. doi: 10.1371/journal.pbio.0040324 (PMC1570498; doi:10.1371/journal.pbio.0040324)
Supplement: Supplementary file 4 [file pbio.0040324.st002.doc]

Table S2. Observed correlation matrix for *Sarracenia* food web. Vol = habitat volume; Prey = prey abundance; Proto = protozoa abundance; Fletch = *Fletcherimyia* (flesh fly larva) abundance; Wyeo = *Wyeomyia* (mosquito larva) abundance; Metrio = *Metriocnemus* (midge larva) abundance; Habro = *Habrotrocha* (rotifer) abundance; Sarra = *Sarraceniopus* (mite) abundance.

|  | Vol | Prey | Proto | Fletch | Wyeo | Metrio | Habro | Sarra |
| --- | --- | --- | --- | --- | --- | --- | --- | --- |
| Vol | 1.000 |  |  |  |  |  |  |  |
| Prey | 0.366 | 1.000 |  |  |  |  |  |  |
| Proto | 0.099 | 0.177 | 1.000 |  |  |  |  |  |
| Fletch | -0.004 | 0.014 | 0.061 | 1.000 |  |  |  |  |
| Wyeo | 0.135 | 0.016 | -0.061 | 0.271 | 1.000 |  |  |  |
| Metrio | 0.432 | 0.156 | 0.211 | 0.113 | 0.228 | 1.000 |  |  |
| Habro | -0.100 | -0.114 | 0.218 | -0.115 | -0.264 | 0.033 | 1.000 |  |
| Sarra | -0.001 | 0.090 | -0.001 | -0.067 | -0.154 | -0.020 | 0.046 | 1.000 |
