# Supplementary material for: Food-Web Models Predict Species Abundances in Response to Habitat Change
Source: PLoS Biol. 2006 Sep 26;4(10):e324. doi: 10.1371/journal.pbio.0040324 (PMC1570498; doi:10.1371/journal.pbio.0040324)
Supplement: Supplementary file 11 [file pbio.0040324.st009.doc]

Table S9. Path structure for top-down + full volume model. Symbols and abbreviations as in Table S3.

|  | Vol | Prey | Proto | Fletch | Wyeo | Metrio | Habro | Sarra |
| --- | --- | --- | --- | --- | --- | --- | --- | --- |
| Vol | s(Vol) | 0 | 0 | 0 | 0 | 0 | 0 | 0 |
| Prey | + | s(Prey) | + | + | 0 | + | 0 | 0 |
| Proto | + | 0 | s(Proto) | 0 | + | 0 | 0 | + |
| Fletch | + | 0 | 0 | s(Fletch) | 0 | 0 | 0 | 0 |
| Wyeo | + | 0 | 0 | + | s(Wyeo) | 0 | 0 | 0 |
| Metrio | + | 0 | 0 | 0 | 0 | s(Metrio) | 0 | 0 |
| Habro | + | 0 | 0 | + | + | 0 | s(Habro) | 0 |
| Sarra | + | 0 | 0 | 0 | 0 | 0 | 0 | s(Sarra) |
